# An Anti-Tumor Vaccine Against Marek's Disease Virus Induces Differential Activation and Memory Response of γδ T Cells and CD8 T Cells in Chickens

**DOI:** 10.3389/fimmu.2021.645426

**Published:** 2021-02-15

**Authors:** Xiaoli Hao, Shuai Li, Jiaqi Li, Yi Yang, Aijian Qin, Shaobin Shang

**Affiliations:** ^1^College of Veterinary Medicine, Yangzhou University, Yangzhou, China; ^2^Institute of Comparative Medicine, Yangzhou University, Yangzhou, China; ^3^Jiangsu Co-innovation Center for Prevention and Control of Important Animal Infectious Diseases and Zoonosis, Yangzhou University, Yangzhou, China; ^4^International Corporation Laboratory of Agriculture and Agricultural Products Safety, Yangzhou University, Yangzhou, China; ^5^Ministry of Education Key Laboratory for Avian Preventive Medicine, Yangzhou University, Yangzhou, China

**Keywords:** γδ T cell, CD8 T cell, immune response, MDV, vaccine

## Abstract

Marek's disease virus (MDV) is a highly oncogenic alphaherpesvirus that causes deadly T-cell lymphomas and serves as a natural virus-induced tumor model in chickens. The most efficacious vaccine, CVI988/Rispens (CVI988), against MD has been used for several decades. However, the mechanisms leading to protective immunity following vaccination are not fully understood. In this study, employing multi-parameter flow cytometry, we performed a comprehensive analysis of T cell responses in CVI988-vaccinated chickens. CVI988 vaccination induced significant expansion of γδ T cells and CD8α^+^ T cells but not CD4^+^ T cells in spleen, lung and blood at early time-points. The expansion of these cells was CVI988-specific as infection with very virulent MDV RB1B did not elicit expansion of either γδ or CD8α^+^ T cells. Phenotypic analysis showed that CVI988 vaccination elicited preferential proliferation of CD8α^+^ γδ T cells and CD8αα co-receptor expression was upregulated on γδ T cells and CD8α^+^ T cells after immunization. Additionally, cell sorting and quantitative RT-PCR showed that CVI988 vaccination activated γδ T cells and CD8α^+^ T cells which exhibited differential expression of cytotoxic and T cell-related cytokines. Lastly, secondary immunization with CVI988 induced the expansion of CD8^+^ T cells but not γδ T cells at higher magnitude, compared to primary immunization, suggesting CVI988 did induce memory CD8^+^ T cells but not γδ T cells in chickens. Our results, for the first time, reveal a potential role of γδ T cells in CVI988-induced immune protection and provide new insights into the mechanism of immune protection against oncogenic MDV.

## Introduction

Marek's disease (MD) is a highly contagious lymphoproliferative disease of chickens caused by Marek's disease virus (MDV) or Gallid herpesvirus 2 (GaHV-2), which serves as a natural virus-induced tumor model in chickens. This disease is characterized by neurological disorders, deadly lymphoma of CD4 T cells and immunosuppression (1). MDV is a member of alpha-herpesviridae subfamily and divided into 3 subfamilies: GaHV-2, GaHV-3, and Meleagrid herpesvirus 1 (MeHV-1 or Herpesvirus of Turkey), corresponding to previous serotypes of MDV-1, MDV-2, and MDV-3, respectively (2, 3). GaHV-2 is oncogenic while GaHV-3 and MeHV-1 are non-oncogenic but all can cause persistent infection. Currently, the commercially available vaccines against MD are CVI988/Rispens (CVI988) from GaHV-2, SB1 strain from GaHV-3, and HVT FC126 strain from MeHV-1 (4–6). The CVI988 is believed to be the most effective vaccine (7) while an equivalent vaccine strain 814 is also widely used in China (8). MD has been successfully controlled worldwide through large-scale vaccination programs, making MD the first oncogenic disease that has been controlled by an effective vaccine (1, 9, 10). However, to date, the mechanisms by which protective immunity is elicited in response to MD vaccines have not been fully revealed (11). In recent years, very virulent plus MDV field strains emerged and broke through the protection elicited by the MD vaccines clinically or experimentally (12–15), highlighting a need for a more efficacious vaccine than CVI988 to combat increasingly virulent MDV strains in the future. However, most of the MD vaccines under development are neither better nor safer than CVI988 (16, 17). Thus, it is crucial to dissect the immune protective mechanisms of current MD vaccines in order to develop better ones against very virulent plus MDV strains.

Early studies showed that sensitized splenocytes by an attenuated MDV strain inhibited plaque formation of MDV-infected leukocytes and chicken kidney cells and killed MD lymphoblastoid cell line (MSB-1) in a T cell-dependent manner (18, 19), suggesting that T lymphocytes play an important role in anti-viral and anti-tumor immunity against MD. More detailed studies showed that cytotoxic T lymphocytes (CTLs) induced by MD vaccines were CD8α^+^ T cells but not CD4^+^ or γδ T cells phenotypically (20, 21). These CTLs can kill reticuloendotheliosis virus (REV)-transformed target cells expressing ICP4, gB, pp38, and meq antigens of MDV (20, 21) as well as many other MDV-encoded antigens (22). Further comparison of the kinetics of CTL activity showed that gB-specific and MHC-restricted CTLs peaked at 8 days post immunization (dpi) in both MD-resistant and -susceptible chickens, but contracted faster in the latter (23). Through neonatal thymectomy and injection of anti-CD4 and -CD8α monoclonal antibodies, Morimura et al. found that depletion of chicken CD4 and CD8 T cells did not increase either tumor incidence or lesion, there was also no significant decrease in the survival rate of CVI988-vaccinated and challenged chickens. However, depletion of CD8α T cells resulted in increased viral titers of MDV within CD4^+^ T cells after challenge (24, 25). Recently, through injection of anti-CD8αβ monoclonal antibodies, Umthong et al. observed that partial depletion of CD8 T cells led to less MD protection and more tumor development in monovalent (SB-1 or HVT) and/or bivalent (SB-1+HVT) -immunized chickens after challenge (26). These studies indicated that CD8T cells participated in anti-viral and anti-tumor immunity to MD. While the cytotoxicity of CD8 T cells has been extensively studied, the dynamic change and magnitude of CD8 T cell response in systemic and local tissues after vaccination have not been determined. In addition, it is unknown whether other T cell subsets respond to MD vaccines.

γδ T cells are innate-like T cells with a restricted TCR repertoire and pre-activated phenotype (27). They can rapidly respond to infection or cytokine stimuli in a non-MHC-restricted manner, secreting a wide range of cytokines and exerting direct cytotoxicity to infected and transformed cells (27). Chickens have a high frequency of γδ T cells that can reach up to 50% of the circulating T cell population (28). Chicken γδ T cells were found to represent a major spontaneously cytotoxic subset that killed LSCC-RP9 cells in a non-MHC-restricted manner (29) and expressed IL-17A (30). During virulent MDV infection, chicken γδ T cells increased in spleens and up-regulated the expression of IFN-γ and IL-10 at different stages of infection (31). However, whether chicken γδ T cells play a role in MD vaccine-induced protection has not yet been studied. In addition, although the expression of cytotoxicity-associated genes including granzyme A, NK-lysin, and perforin as well as T helper cytokine IFN-γ have been detected after vaccination with CVI988 (32, 33), it is unclear which subset of immune cells contribute to the expression of specific cytokines or effector molecules.

In this study, employing multi-parameter flow cytometry, we carried out a comprehensive analysis of T-cell immunity in CVI988-vaccinated chickens in order to dissect the mechanism of immune protection elicited by the CVI988. We found CVI988 vaccine induced significant expansion of γδ T cells and CD8α^+^ T cells in spleen, lung and blood at early time-points after immunization. CVI988-activated γδ T cells and CD8α^+^ T cells displayed phenotypic and dynamic changes and expressed cytokine profiles differentially. However, γδ T cells did not exhibit a recall response to secondary immunization with CVI988. These results increase our understanding of the mechanism of immune protection against MD.

## Materials and Methods

### Ethics Statement

All animal experiments were approved by Jiangsu Province Administrative Committee for Laboratory Animals (Permission number: SYXK-SU-2017-0007) and complied with the guidelines of Jiangsu Province Laboratory Animal Welfare and ethics of Jiangsu Province Administrative Committee of Laboratory Animals.

### Chicken, Virus, Vaccine, and Antibodies

One-day-old specific-pathogen-free (SPF) White Leghorn chickens were purchased from Zhejiang Lihua Agricultural Technology Co., Ltd. (Ningbo, China). MDV strain RB1B was provided by Dr. Aijian Qin (Yangzhou University) and was used for infection of chickens. CVI988/Rispens, a commercially available MD vaccine, was purchased from Boehringer Ingelheim (Shanghai, China). The virus stocks were titrated and stored in liquid nitrogen. Monoclonal antibodies (mAb) specific for chicken CD3 (CT-3), CD8α (CT-8), CD8β (EP42), TCRγδ (TCR-1), CD4 (CT-4) with different fluorochrome conjugates were purchased from SouthernBiotech (Birmingham, AL, USA) (Table 1).

**Table 1:** Antibodies used for flow cytometry in this study.

**Marker**	**Clone**	**Isotype**	**Conjugate**	**Source**
CD3	CT-3	mouse IgG1	PerCP-Cy5.5	Southern Biotech
TCRγδ	TCR-1	mouse IgG1	Biotin	Southern Biotech
CD8α	CT-8	mouse IgG1	AF700	Southern Biotech
CD8β	EP42	mouse IgG2a	PE	Southern Biotech
CD4	CT-4	mouse IgG1	Pacific blue	Southern Biotech
Streptavidin	—	—	BV510	BioLegend
Dead Cell Stain	—	—	FVD eFluor 780	Thermo Fisher Scientific

### Animal Experiments

One-day-old SPF White Leghorn chickens were randomly divided into three groups (24 chickens each group in one separate isolator). One group of chickens was vaccinated on day 1 post-hatch via intra-abdominal route with 2,000 plaque forming units (PFU) of CVI988 (the vaccinated group). The second group of chickens was infected with 1,000 PFU of virulent strain RB1B (the infection group). The third group received PBS and served as control. Six birds per group were euthanized by CO_2_ inhalation and peripheral blood, lungs and spleens were collected at 3, 7, 14, and 21 dpi for virus quantification and single cell isolation. For the group infected with virulent MDV strain RB1B, we only harvested tissue samples at 3, 7, and 14 dpi due to insufficient number of birds at later time-points. Each sample at 3 and 7 dpi was pooled from two birds.

### Single Cell Preparation

Single cell suspension from spleens and lungs were prepared as previously described (34) and peripheral blood mononuclear cells (PBMC) were isolated from blood as per previous report (35). Briefly, spleens were mechanically disrupted and pushed through a 70 μm nylon cell strainer and the cells were resuspended in 10 ml with phosphate buffered saline (PBS) containing 2% fetal bovine serum (FBS). Lungs were cut into small pieces and digested with collagenase IV (1 mg/ml; Sigma, St. Louis, MO, USA) and DNase I (30 μg/ml; Sigma) for 30 min at 37°C before disruption. Cell suspensions were overlaid onto Histopaque-1077 (Sigma-Aldrich, Poole, UK) at a 1:1 ratio to isolate mononuclear cells. After centrifugation at room temperature for 30 min at 400 g, the interface was collected and added to 10 mL complete medium(CM; RPMI-1640 supplemented with 10% FBS (Gibco, Grand Island, NY, USA), 1% penicillin plus streptomycin [Invitrogen, Carlsbad, CA, USA)] and washed with PBS containing 2% FBS. To isolate PBMCs, whole blood containing anti-coagulant heparin sodium was diluted with equal volume of PBS and layered on histopaque-1077 and subjected to the above protocol. Cells were counted using a hemocytometer and the final cell density was adjusted to 2 × 10^7^ cells/mL.

### Flow Cytometry and Cell Sorting

Cells were plated in 96-well V-bottom plates with each well containing 2 × 10^6^ cells in 100 μl FACS buffer (PBS containing 0.5% BSA). After centrifugation, cells were first incubated with chicken serum to block FC receptors, and then stained with 50 μl of antibody cocktail containing anti-chicken CD3, CD8α, CD8β, TCRγδ, and CD4 antibody for 20 min at room temperature. The cells were stained with fixable viability dye (FVD) eFluor 780 (Thermo Fisher Scientific, Waltham, MA, USA) for excluding dead cells. The cells were washed twice with FACS buffer by spinning down at 400 g for 5 min at 4°C. A minimal number of 100,000 cells was acquired for FACS analysis. Flow cytometry was performed with a FACS LSRFortessa (BD Biosciences, Franklin Lakes, NJ, USA) and the data were analyzed by FlowJo software (Tree Star Inc., Ashland, OR, USA). Percentages and absolute numbers were subsequently calculated.

For cell sorting, splenocytes (2–5 × 10^7^ cells) were stained with mouse anti-chicken CD3, TCRγδ, and CD8α mAb for 30 min on ice and then washed twice in FACS buffer by centrifugation at 400 g for 5 min at 4°C. Sorting was performed on a FACSAria SORP cytometer (BD Biosciences, Franklin Lakes, NJ, USA). Two populations (CD3^+^TCRγδ^+^ and TCRγδ^−^CD3^+^CD8a^+^) were sorted and collected into round-bottom polystyrene tubes with complete medium. The purity of the two subsets was >96.8%. The isolated cells were either directly used or stored at −80°C until further analysis.

### Quantitative PCR and RT-PCR

For absolute quantification of MDV/CVI988 genome loads, we employed real-time quantitative duplex PCR (q-PCR) for the detection of MDV/CVI988 meq gene from blood, spleen and lung. The chicken ovotransferrin (ovo) was used as a reference gene as previously described (36). Total cellular DNA was extracted using the High Pure PCR Template Preparation Kit (Roche, Mannheim, Germany) according to the manufacturer's instructions. The q-PCR was performed using a Light Cycler 480 instrument (Roche, Mannheim, Germany) and AceQ U^+^ Probe Master Mix (Vazyme, Nanjing, China). A final volume of 20 μl reaction consisted of 10 μl master mix, 1 μl forward primer, 1 μl reverse primer, 6 μl PCR-grade water, and 2 μl of target DNA template. The thermal cycling program was: initial denaturation at 95°C for 30 s, followed by 40 cycles of denaturation at 95°C for 5 s and annealing/extension at 60°C for 30 s, with end point melt-curve analysis. Recombinant pCR2.1 vector (Promega, USA) carrying *meq* and *ovo* gene was serially diluted 10-fold and used for generating a standard curve in order to calculate the absolute copy number of the genes.

For quantification of cytokine expression, the primers used for each cytokine and housekeeping gene are summarized in Table 2. Total RNA was isolated from mononuclear cells or sorted cells from spleen and lung with FastPure Cell/Tissue Total RNA Isolation Kit (Vazyme, Nanjing, China). The RNA was reverse transcribed into cDNA with HiScript III RT SuperMix for qPCR according to the manufacturer's instruction (Vazyme, Nanjing, China). The SYBR green based real-time PCR was performed with ChamQ Universal SYBR qPCR Master Mix (Vazyme, Nanjing, China). The relative fold change of target genes was calculated by 2^−ΔΔCT^ method. The Ct value for each sample was normalized to housekeeping gene chicken β-actin.

**Table 2:** Primers sequences for real-time PCR.

**Target gene**	**Primer name**	**Primer sequence (5^**′**^-3^**′**^)**	**Accession number**
Granzyme A	Granzyme A forward Granzyme A reverse	CCTGATACTTCCTGGAGATTTGTGC TTTTTGTCCGTGAATGGGCTC	NM_204457.1
Perforin	Perforin forward Perforin reverse	CACCCGCACCAAAAGATGAAG CGCCTCCTGGAAAACACACAAC	KC551799.1
IFN-γ	IFN-γ forward IFN-γ reverse	CTCCCGATGAACGACTTGAG CTGAGACTGGCTCCTTTTCC	FJ977575.1
TNF-α	TNF-α forward TNF-α reverse	CGCTCAGAACGACGTCAA GTCGTCCACACCAACGAG	MF000729.1
IL-2	IL-2 forward IL-2 reverse	TTGGCTGTATTTCGGTAGCA GTGCACTCCTGGGTCTCAGT	NM_204153.1
NK lysin	NK lysin forward NK lysin reverse	GATGGTTCAGCTGCGTGGGATGC CTGCCGGAGCTTCTTCAACA	DQ186291
IL-17A	IL-17 forward IL-17 reverse	CTCCTCTGTTCAGACCACTGC ATCCAGCATCTGCTTTCTTGA	AJ493595.1
IL-10	IL-10 forward IL-10 reverse	AGCAGATCAAGGAGACGTTC ATCAGCAGGTACTCCTCGAT	NM_001004414.2
β-actin	β-actin forward β-actin reverse	CCAACTGGGATGATATGGAGAAG AGGCATACAGGGACAGCACA	NM205518

### Statistical Analyses

Statistical significance was determined with the independent-samples *t*-test in the SPSS statistics software (IBM company, SPSS 23.0). Data are presented as a comparison between the control and the infected groups. Results were considered to be statistically significant if *p* < 0.05 (^*^), *p* < 0.01 (^**^), or *p* < 0.001 (^***^).

## Results

### Dynamics of Productive Infection in Chickens Caused by CVI988

**Figure 1:**
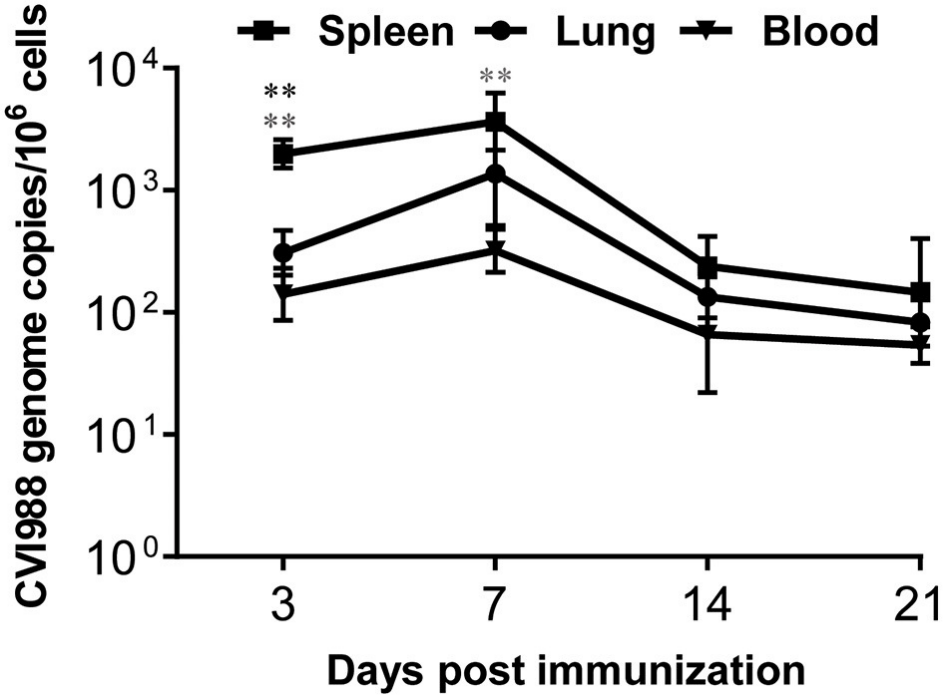
The quantification of CVI988 genome copies in chicken tissues after immunization. The meq/ovo duplex PCR was performed using DNA samples extracted from mononuclear cells isolated from spleen, lung, and blood of the immunized chickens at 3, 7, 14, and 21 days post-immunization (dpi). Using the standard curves for the meq and ovo reactions, genome copy number per million host cells was determined. Data presented are the means ± SD for six vaccinated chickens. *, compared to the CVI988 genome copies from spleen. (*P < 0.05; **P < 0.01; ***P < 0.001).

Similar to virulent MDV strains, the current MDV vaccine strains are known to establish persistent infection after vaccination (1). To confirm that the immunized chickens are indeed productively infected, we quantified CVI988 genome copy numbers by detecting *meq* gene expression in leucocytes isolated from spleen, lung and blood at 3, 7, 14, and 21 dpi. As shown in Figure 1, the expression of *meq* gene was detected in all the organs from the vaccinated chickens at all the time-points, and the copy number of *meq* gene peaked at 7 dpi, suggesting CVI988 vaccine caused a productive infection in the 1st week, consistent with a previous report (37). In addition, the *meq* copy number in the spleen was significantly higher than in lung (*p* < 0.01) and blood (*p* < 0.01) at 3 dpi and in blood (*p* < 0.01) at 7 dpi (Figure 1). All samples from the unimmunized group were *meq* negative (data not shown).

### CVI988 Induces Significant Expansion of γδ T Cells and CD8α^+^ T Cells but Not CD4^+^ T Cells After Immunization

**Figure 2:**
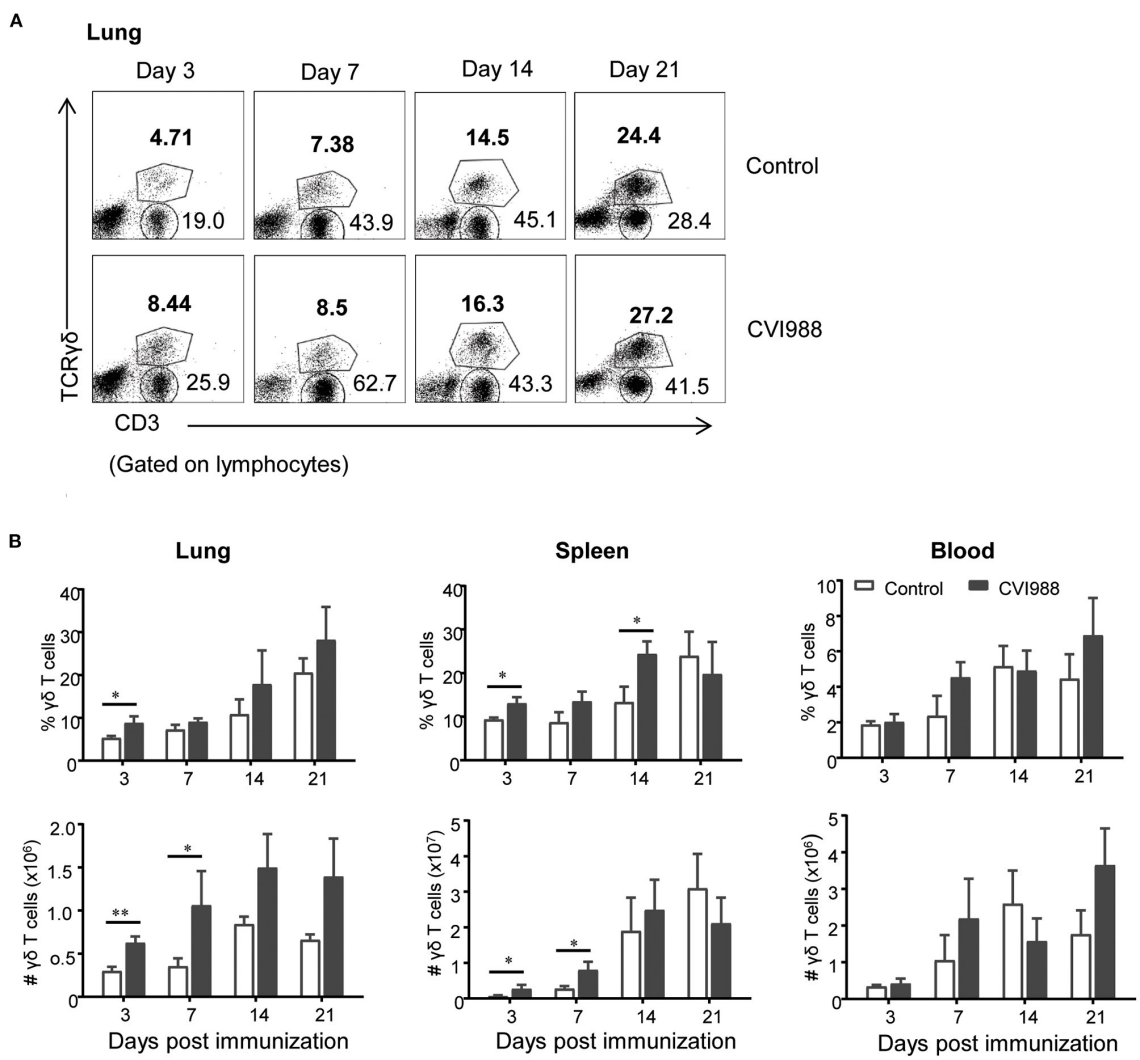
The dynamic changes of chicken γδ T cells after CVI988 immunization. Chickens were immunized with or without CVI988 (2,000 PFU) and euthanized at indicated time-points, single cell suspensions from the lung, spleen, and blood were prepared for numerating γδ T cells by flow cytometry. **(A)** Representative dot-plots depict γδ T cells and TCRγδ^−^CD3^+^ T cells in the lungs of indicated birds at 3, 7, 14, and 21 dpi. Numbers indicate the percentages of γδ T and CD3^+^ T cells. **(B)** Kinetic changes of the percentages (upper panel) and total numbers (lower panel) of γδ T cells in the lung, spleen and blood of non-vaccinated and CVI988-vaccinated chickens. Data shown are mean ± SD from six birds per group. *P < 0.05, **P < 0.01.

Cell-mediated immunity is thought to play dominant roles in protection against MD. However, the dynamic changes of distinct T cell subsets in the periphery and local tissues of chicken have not been well-characterized after vaccination with CVI988 (11). We therefore employed multi-parameter flow cytometry to analyze the changes of γδ T cells, CD4, and CD8 T cells in spleen, lung, and blood at 3, 7, 14, and 21 dpi which corresponds to the phases of cytolytic replication, latent and transformation of MDV infection. A basic gating strategy is shown in Supplementary Figure 1. As depicted in representative dot-plots (Figure 2A; Supplementary Figure 2), the percentage of γδ T cells and TCRγδ^−^CD3^+^ T cells increased in the lungs of unimmunized chickens, along with the development of immunocompetence of chickens from 3-day old to 21-day old (38). Similar findings were observed in the spleen and blood (data not shown). However, compared to the control, CVI988 vaccination induced significantly higher percentage and absolute number of γδ T cells in the spleens and lungs at 3 and/or 7 dpi but not in the blood at any time-point (Figure 2B). There is a trend toward higher number of splenic and lung γδ T cells at 14 and 21 dpi in the vaccinated birds but no statistical difference was found between the two groups (Figure 2B).

**Figure 3:**
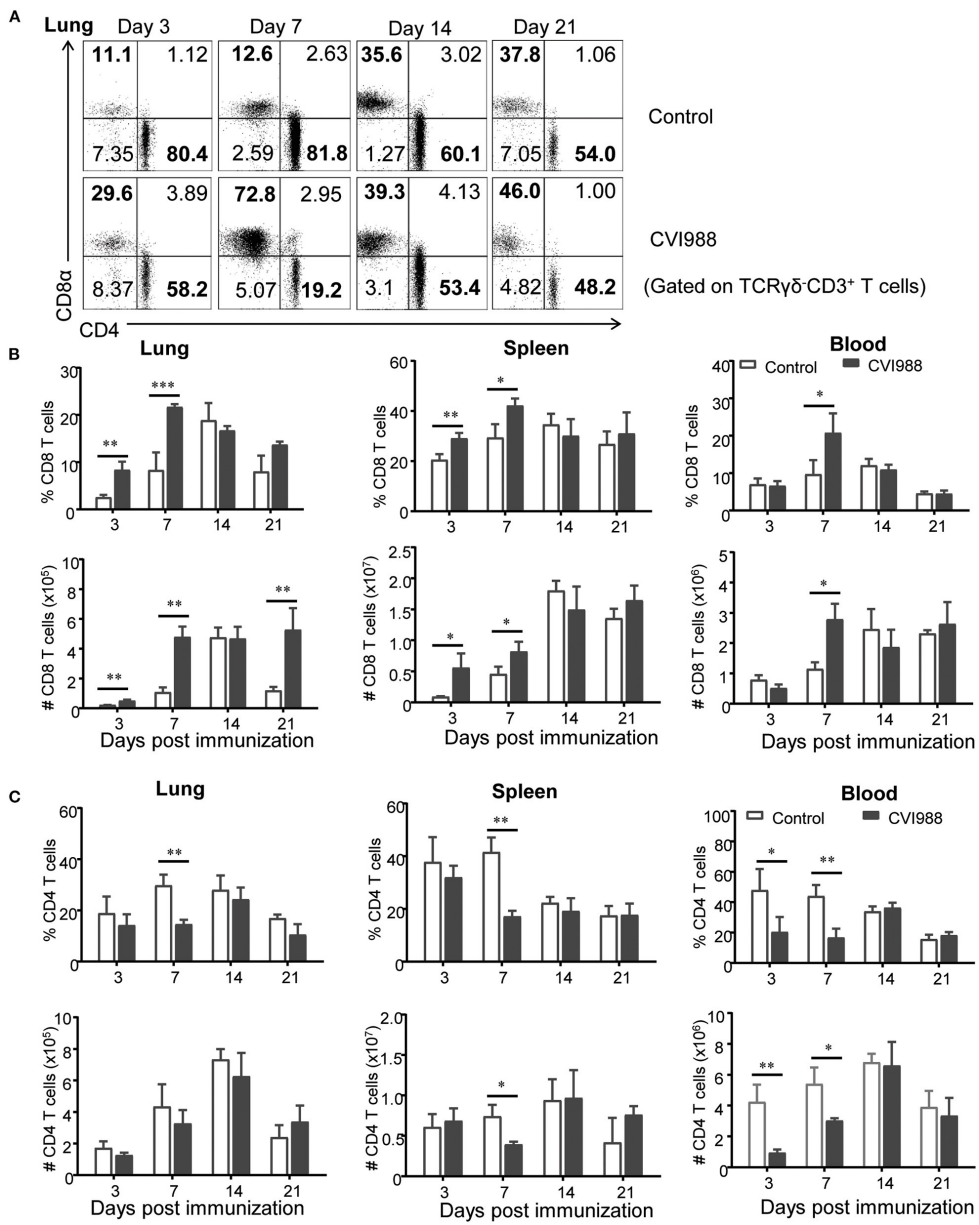
The dynamic changes of CD8^+^ and CD4^+^ T cells in chickens after CVI988 immunization. Chickens were immunized with or without CVI988 and euthanized at indicated time points, single cell suspensions from lung, spleen and blood were prepared for counting CD8^+^ and CD4^+^ T cells by flow cytometry. **(A)** Representative dot-plots depict CD8^+^ and CD4^+^ T cells (TCRγδ^−^CD3^+^) in the lungs of CVI988-immunized (CVI988) and non-vaccinated birds (control) at 3, 7, 14, and 21 dpi. Numbers represent the percentages of cells in each quadrant. **(B)** Kinetic changes of the percentage (upper panel) and total number (lower panel) of CD8^+^ T cells in the lung, spleen, and blood of non-vaccinated and CVI988-vaccinated chickens. **(C)** Kinetic changes of the percentage (upper panel) and total number (lower panel) of CD4^+^ T cells in the lung, spleen and blood of non-vaccinated and CVI988-vaccinated chickens. Data shown are mean ± SD from six birds per group. *P < 0.05, **P < 0.01, ***P < 0.001.

While TCRγδ^−^CD3^+^ T cells increased at higher magnitude in the CVI988-vaccinated chickens, compared to the control (Figure 2A), it was unclear whether there was difference in the dynamic changes of distinct T cell subsets between the two groups. We defined three T cell subsets (CD8α^+^, CD4^+^, and CD4^−^CD8α^−^) among total TCRγδ^−^CD3^+^ T cells (shown in Figure 3A) and found that CVI988 vaccination elicited significantly higher percentage and number of CD8α^+^ T cells (TCRγδ^−^CD3^+^CD8α^+^) in the spleens and lungs at 3 and 7 dpi and in the blood at 7 dpi, compared to the control (Figure 3B). There was no difference in the percentage and number of CD8α^+^ T cells in the spleen, lung and blood at 14 and 21 dpi between the two groups except a significantly higher number of CD8α^+^ T cells in the lungs of the immunized chicken at 21 dpi (Figure 3B). Interestingly, CVI988 immunization did not induce CD4^+^ T cells expansion; instead a significant reduction was detected in the percentage and number of CD4^+^ T cell in the spleens and lungs at 7 dpi and at 3 and 7 dpi in the blood, compared to the control (Figure 3C). These results suggested that CVI988 vaccine induced significant expansion of γδ T cells and CD8α^+^ T cells but not CD4^+^ T cells in lung and spleen at the early time-points after immunization.

To determine if infection with the very virulent MDV strain RB1B also induced expansion of γδ T cells and CD8α^+^ T cells in chicken, we examined the dynamic changes of these populations in the spleen and lung of RB1B-infected birds. We found that the MDV RB1B strain transiently induced higher percentage of γδ T cells in the lung at 7 dpi but not in the spleen at any time-point and no significant expansion of CD8α^+^ T cells in spleen and lung over the course of infection, compared to mock-infected birds (Supplementary Figure 3). These findings suggested that the expansion of γδ T cells and CD8α^+^ T cells at early time-points after CVI988 vaccination might be a CVI988-specific immune response.

### CVI988 Vaccination Elicits Preferential Proliferation of CD8α^+^ γδ T Cells

**Figure 4:**
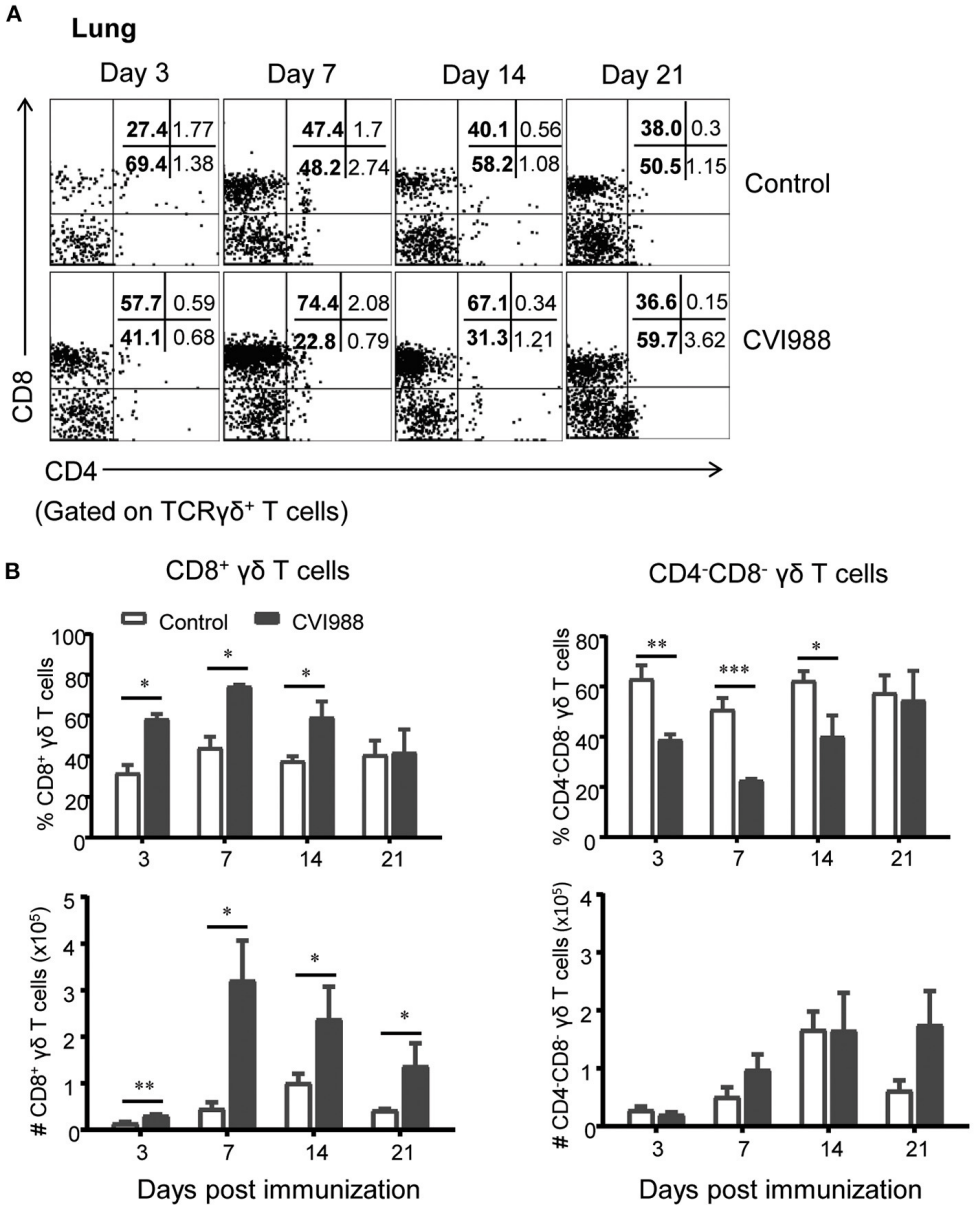
The preferential proliferation of CD8α^+^ γδ T cells after CV988 vaccination. Single cell suspensions were prepared from the control and CVI988-immunized chickens at indicated time-points and the dynamic changes of different subsets of γδ T cells (TCRγδ^+^CD3^+^) were analyzed by flow cytometry. **(A)** Representative dot-plots depict different subsets of γδ T cells (CD8^+^, CD4^+^, and CD4^−^CD8^−^) in the lung of indicated birds at 3, 7, 14, and 21 dpi. Numbers represent the percentages of cells in each quadrant. **(B)** Kinetic changes of the percentage (upper panel) and number (lower panel) of CD8^+^ (left) and CD4^−^CD8^−^ γδ T cells (right) in the lungs of the control and CVI988-vaccinated chickens. Data shown are mean ± SD from six birds per group. *P < 0.05, **P < 0.01, ***P < 0.001.

Previous studies demonstrated that γδ T cells of chicken can be divided into three distinct subsets: CD8^+^, CD4^+^, and CD4^−^CD8^−^double negative (DN) (39). Therefore, we evaluated the changes of these γδ T cell subsets after CVI988 immunization. We found that among total γδ T cells, DN γδ T cells were most dominant in the lung (Figure 4A) and CD8^+^γδ T cells were most dominant in the spleen of un-immunized chicken (Supplementary Figure 4). After CVI988 immunization, CD8^+^γδ T cells in the lung and spleen significantly increased at early time-points while DN γδ T cells decreased in terms of percentage (Figure 4A; Supplementary Figure 4). Compared to the un-immunized chickens, the vaccinated birds had a significantly higher percentage and number of CD8^+^γδ T cells in the lung at all the time-points (Figure 4B) and in the spleen at 3, 7 dpi (Supplementary Figure 4). There was no difference in the numbers of DN γδ T cells in the lung and spleen between the two groups though the percentage of DN γδ T cells was decreased (Figure 4B; Supplementary Figure 4). Additionally, there was no difference in the percentage of CD4^+^γδ T cells between the two groups (data not shown). These results indicated that CVI988 vaccination elicited preferential proliferation of CD8α^+^γδ T cells.

### CD8αα Co-receptor Expression Is Upregulated on γδ T Cells and CD8α^+^ T Cells After Immunization

**Figure 5:**
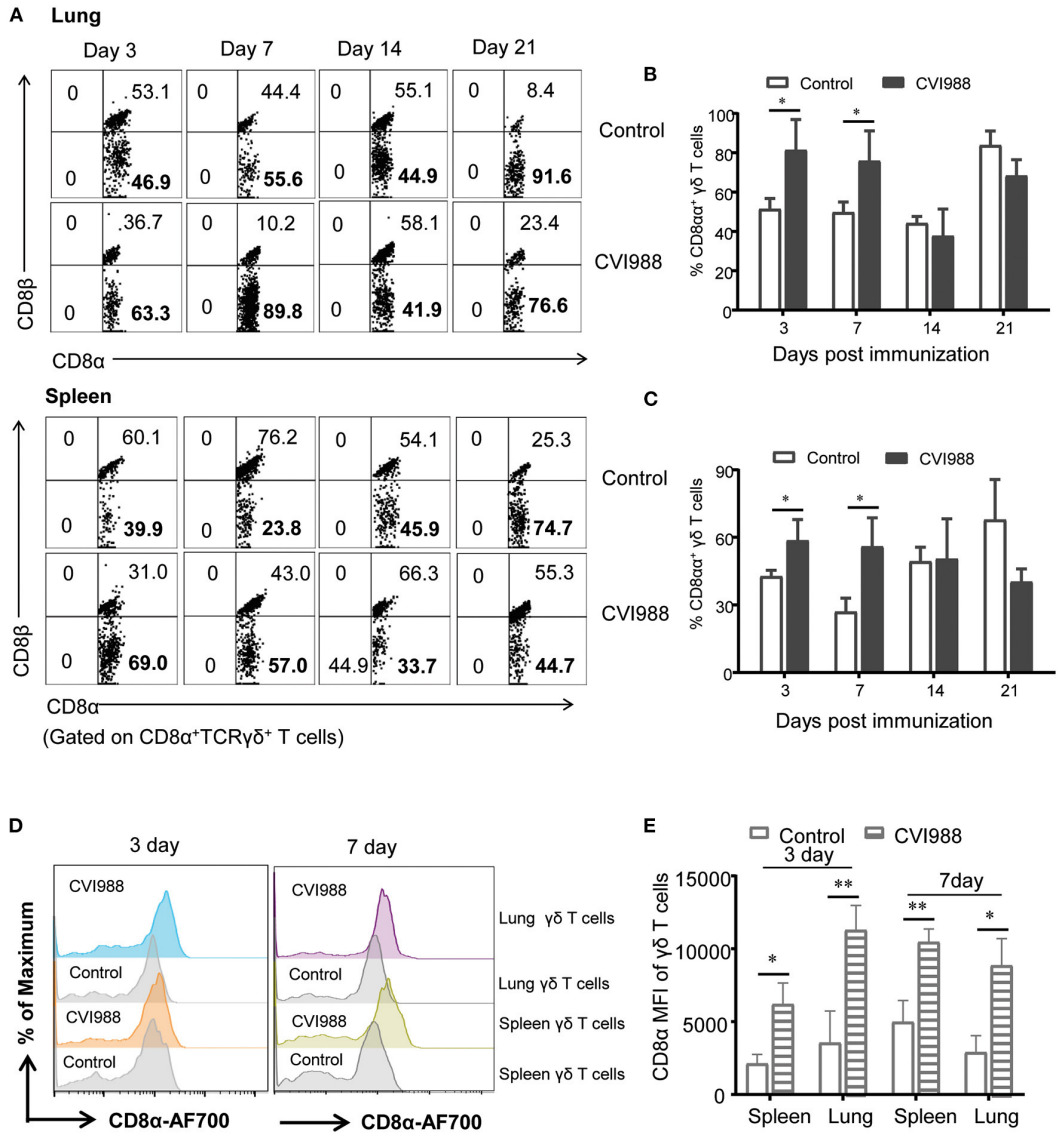
The upregulated expression of CD8αα co-receptor on γδ T cells after immunization. Single cell suspensions were prepared from the lungs and spleens of mock- and CVI988-immunized chickens at indicated time-points, CD8α and CD8β expressions on the gated CD8α^+^γδ T cells were analyzed by flow cytometry. **(A)** Representative dot-plots depict CD8αβ^+^ and CD8αα^+^ (CD8α^+^CD8β^−^) γδ T cell populations from the two groups at 3, 7, 14, and 21 dpi. Numbers represent the percentages of cells in each quadrant. **(B,C)** Kinetic changes of the percentage of CD8αα^+^γδ T cells in the lung **(B)** and spleen **(C)** of non-vaccinated and CVI988-vaccinated chickens at indicated time-points. **(D)** Representative histograms show CD8α expression on γδ T cells from the lung and spleen of non-vaccinated and CVI988-vaccinated chickens at 3 and 7 dpi. **(E)** Bar graphs show geometric mean fluorescence intensity (MFI) of CD8α on γδ T cells from the two groups at 3 and 7 dpi. Data shown are mean ± SD from six birds per group. *P < 0.05, **P < 0.01.

In mammals, CD8^+^ T cells and γδ T cells or innate-like T cells can express two forms of CD8 coreceptor: CD8αα homodimer and CD8αβ heterodimer (40). We therefore examined the differential expression of these two coreceptors on chicken γδ T cells and CD8α^+^ T cells (TCRγδ^−^CD3^+^CD8α^+^). The results showed that CD8αα^+^ γδ T cells (CD8α^+^CD8β^−^) but not CD8αβ^+^γδ T cells (CD8α^+^CD8β^+^) proportionally increased at the early time-points after CVI988 vaccination (Figure 5A). The vaccinated chickens had a significantly higher percentage of CD8αα^+^γδ T cells in spleen and lung at 3 and 7 dpi but not in the blood, compared to the control (Figures 5B,C). This was consistent with the trend of the expansion of γδ T or CD8α^+^γδ T cells. In addition to the increase of the frequency of CD8αα^+^γδ T cells, the mean fluorescence intensity (MFI) of CD8α was increased on the CD8α^+^γδ T cells of the CVI988-vaccinated birds, suggesting CVI988 immunization upregulated the expression of CD8α at the level of a single cell (Figures 5D,E). Similar to γδ T cells, the frequency of CD8αα^+^CD8^+^ T cells (TCRγδ^−^CD3^+^CD8α^+^) also increased in the vaccinated chickens and the expression of CD8α on this population is also upregulated in spleens and lungs at 3 and 7 dpi but to a lesser extent compared to γδ T cells (Supplementary Figure 5).

### γδ T Cells and CD8α^+^ T Cells Exhibit Differential Cytokine Expression Upon CVI988 Vaccination

**Figure 6:**
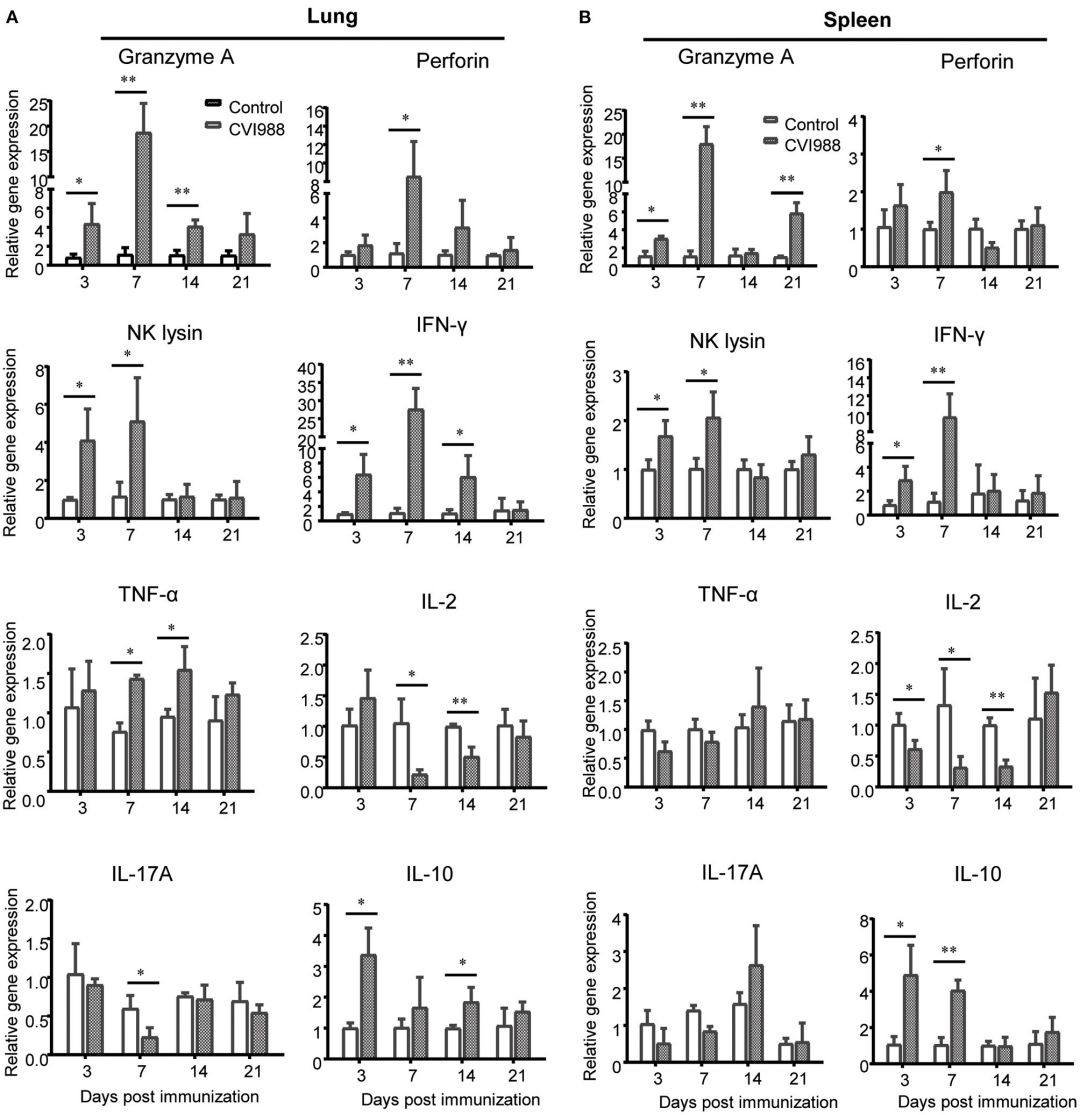
Relative quantification of cytotoxic and T cells-related effector molecules in the lung and spleen after CVI988 immunization. Total RNA was extracted from single cell suspensions isolated from the lungs **(A)** and spleens **(B)** of mock- (control) and CVI988-immunized (CVI988) chickens and then cDNA was prepared by reverse transcription. The mRNA expression of cytokines related to cytotoxicity and effector T cells including granzyme A, perforin, NK lysin, IFN-γ, TNF-α, IL-2, IL-17A, and IL-10 were quantified using SYBR green based real-time PCR assays at 3, 7, 14, and 21 dpi, respectively. The data were collected from three biological samples in each group, each sample was performed in triplicate for qPCR. Data were normalized against β-actin and expressed as the mean ± SD. *P < 0.05, **P < 0.01, ***P < 0.001.

The profiles of cytokine expression related to innate immunity in the context of MDV infection or vaccination were extensively examined in previous studies (32, 33, 41). However, the expression of cytotoxic and T cell-related effector molecules has not been well-characterized. To address this issue, we chose genes related to cytotoxicity and effector T cell function such as *granzyme A, perforin, NK lysin, IFN-*γ, *TNF-*α, *IL-2, IL-17A*, and *IL-10*, and quantified their relative gene expression in leucocytes isolated from the spleens and lungs of vaccinated and unvaccinated chicken. We found that the mRNA expression of *granzyme A, perforin, IFN-*γ, and *NK lysin* was significantly upregulated in spleen and lung at the early phase and peaked at 7 dpi, with higher expression in the lung (Figures 6A,B). *TNF-*α mRNA expression increased in the lung at 7 and 14 dpi and there was no difference in the expression of *IL-17A* mRNA. On the other hand, *IL-2* mRNA expression was down-regulated (Figures 6A,B). There was a significant increase in the expression of *IL-10* mRNA in the lung at 3, 14 dpi and in the spleen at 3, 7 dpi, compared to the control (Figures 6A,B).

**Figure 7:**
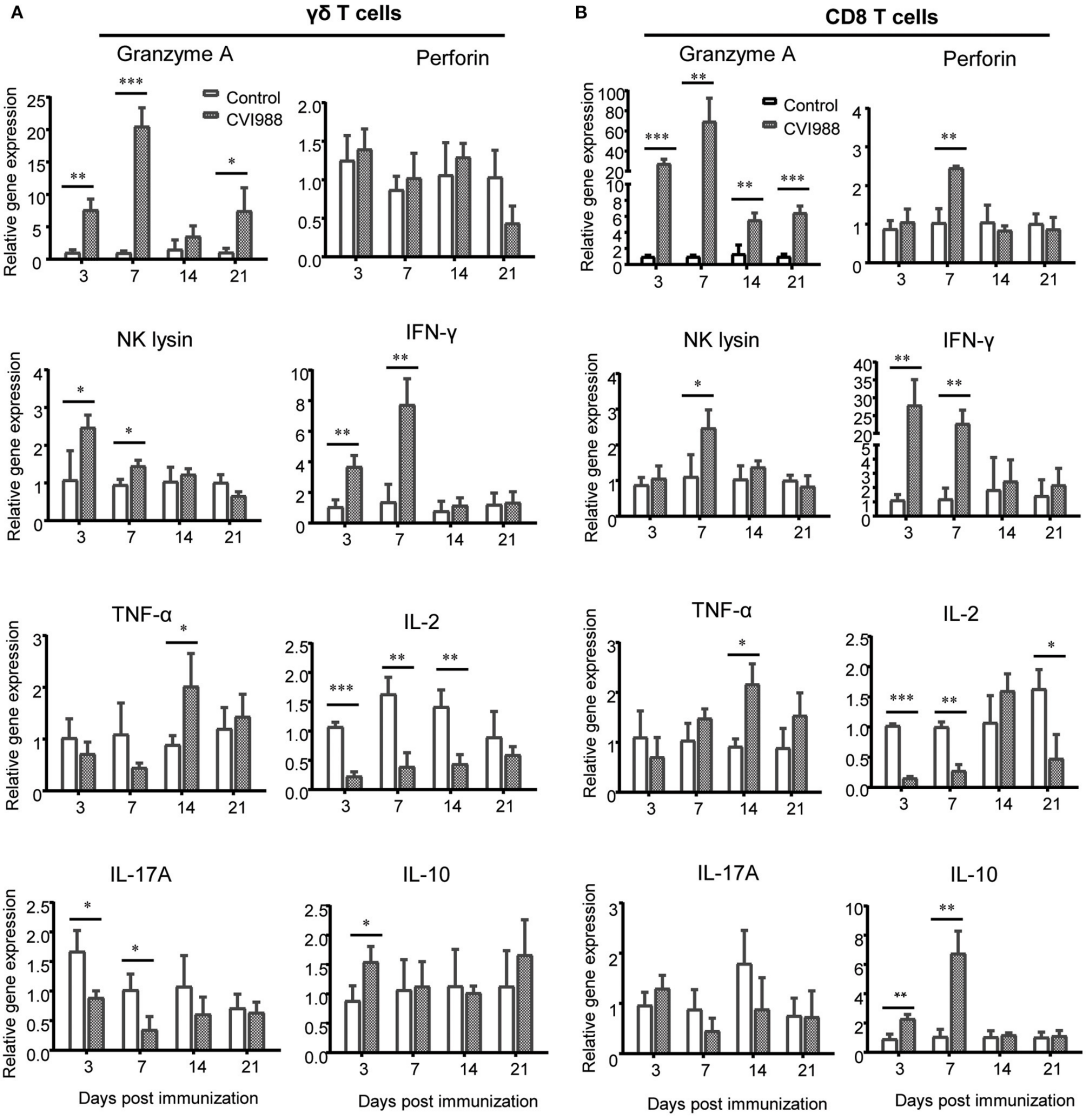
The cytokine profiles expressed by sorted γδ T cells and CD8α^+^ T cells. Single cell suspensions were prepared from the lungs and spleens of mock- and CVI988-immunized chickens and stained with antibodies against chicken TCRγδ, CD8α, and CD3. **(A)** γδ T cells (TCRγδ^+^CD3^+^) and **(B)** CD8α^+^ T cells (TCRγδ^−^CD3^+^CD8α^+^) were sorted by flow cytometry and subjected to quantitative RT-PCR for mRNA expression of cytokines related to cytotoxicity and effector T cells including granzyme A, perforin, NK lysin, IFN-γ, TNF-α, IL-2, IL-17A, and IL-10 at 3 (*n* = 9), 7 (*n* = 9), 14 (*n* = 4), and 21 dpi (*n* = 4). Data were normalized against β-actin and expressed as the mean ± SD from four to nine birds per group. *P < 0.05, **P < 0.01, ***P < 0.001.

To determine which subset of immune cells expressed specific cytokines or effector molecules, we sorted γδ T cells and CD8α^+^ T cells by flow cytometry from the immunized and unimmunized chicken and quantified the transcriptional expression of those aforementioned cytokine genes by qPCR. The results showed that mRNA expression of *Granzyme A, IFN-*γ and *NK lysin* but not *Perforin* in sorted γδ T cells was significantly increased at 3 and 7 dpi, *TNF-*α mRNA expression increased at 14 dpi and *IL-10* mRNA expression increased at 3 dpi whereas the mRNA expression of *IL-2* and *IL-17A* was down-regulated (Figure 7A). Similar to γδ T cells, sorted CD8α^+^ T cells also exhibited increased expression of *Granzyme A, IFN-*γ, *NK lysin, TNF-*α, and *IL-10* and decreased *IL-2* expression after CVI988 immunization, but with different kinetics for the expression of *Granzyme A, NK lysine*, and *IL-10* and with higher expression levels for *Granzyme A* and *IFN-*γ, compared to γδ T cells (Figure 7B). Unlike γδ T cells, CD8α^+^ T cells increased *perforin* expression at 7 dpi and there was no change for *IL-17A* expression in CD8α^+^ T cells between the two groups (Figure 7B). These results indicated that activated γδ T cells and CD8α^+^ T cells after CVI988 vaccination expressed cytokine profiles differentially.

### Recall Response of Chicken γδ T and CD8α^+^ T Cells Upon Secondary Immunization

**Figure 8:**
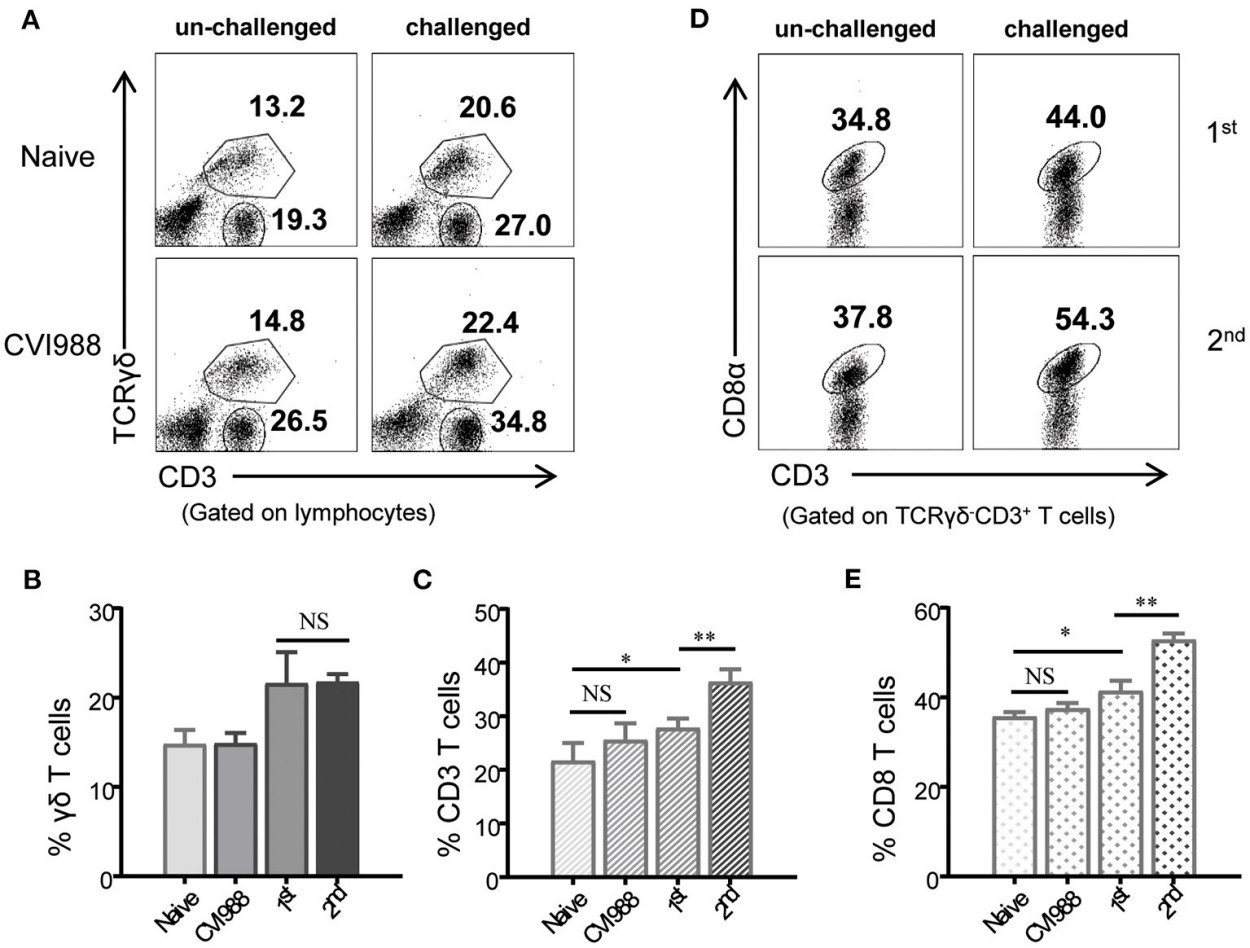
Recall response of chicken γδ T and CD8α^+^ T cells after secondary immunization. Chickens were immunized with or without CVI988 vaccine and rested for 4 weeks, then the immunized and age-matched naïve chickens were challenged with or without CVI988. The primary and secondary response of γδ T cells, CD3^+^, and CD8α^+^ T cells (TCRγδ^−^CD3^+^CD8α^+^) in naïve and previously-immunized chickens were compared by flow cytometry. **(A)** Representative dot-plots depict γδ T cells and CD3^+^ T cells in the lungs of indicated birds challenged with or without CVI988. Numbers indicate the percentages of γδ T and CD3^+^ T cells. **(B,C)** Bar graphs show the percentages of γδ T **(B)** and CD3^+^ **(C)** T cells in the lungs of indicated chickens at 7 days post-challenge. **(D)** Representative dot-plots depict CD8α^+^ T cells among total CD3^+^ T cells in the lungs of indicated birds challenged with or without CVI988. Numbers indicate the percentages of CD8α^+^ T cells. **(E)** Bar graph shows the percentage of CD8α^+^ T cells in the lungs of indicated chickens at 7 days post-challenge. Data shown are mean ± SD from four birds per group. *P < 0.05, **P < 0.01, NS, no statistical difference.

Studies in mammals have shown that compared to naïve T cells, memory T cells proliferate faster and differentiate into secondary effector T cells at higher magnitude upon re-infection (42–44). To examine whether chicken γδ T cells had memory response after secondary immunization, chickens were immunized with or without CVI988 vaccine and rested for 4 weeks. The immunized chickens and age-matched naïve chickens were then challenged with or without CVI988. The primary and secondary response of γδ T, CD3^+^, and CD8α^+^ T cells (TCRγδ^−^CD3^+^) in naïve and previously immunized chickens were compared. As shown in Figure 8, there was no difference in the frequency of γδ T and CD3^+^ T cells between naïve and previously immunized chickens before challenge. After challenge with CVI988, both primary and secondary γδ T cells significantly increased, however, there is no difference between the two groups (Figures 8A,B). In contrast, secondary TCRγδ^−^CD3^+^ T cells expanded faster than primary TCRγδ^−^CD3^+^ T cells after challenge (Figures 8A,C). Further comparison of the primary and secondary response of T cell subsets confirmed that it was CD8α^+^ but not CD4^+^ T cells (TCRγδ^−^CD3^+^) contributed to the total memory CD3^+^ T cell response (Figures 8D,E). These findings suggested that chicken γδ T cells did not possess a recall response to secondary immunization with CVI988 whereas CD8α^+^ T cells had memory response.

## Discussion

MDV is a strictly cell-associated virus and thus, cell-mediated immunity is thought to be of paramount importance against MD (11). Although the roles of T lymphocytes and cytotoxic CD8 T cells as well as early cytokine expression in MD vaccine-induced protection have been extensively examined in previous studies (18–26, 32, 33, 41), there were a lot of gaps in the knowledge of T-cell immunity against MD (11). For instance, [1] what are the dynamic and phenotypic changes of T cells responses in systemic and local tissues after vaccination? [2] whether, T cell subsets other than CD8 T cells respond to MD vaccination? [3] In addition to cytotoxicity, whether T cells express specific cytokines and effector molecules that may correlate with immune protection induced by MD vaccines? In the present study, we filled some of these gaps through a more comprehensive analysis of T cell responses to CVI988 vaccination by flow cytometry. Our results revealed a potential role of γδ T cells in CVI988-induced immune protection in chickens. In addition, our results also demonstrated that γδ T cells and CD8α^+^ T cells displayed phenotypic and functional differences and changes after CVI988 vaccination characterized by preferential proliferation of CD8α^+^ γδ T cells, upregulation of CD8ααco-receptor on both γδ T cells and CD8α T cells, differential cytokine expression profiles and distinct memory response to secondary immunization.

MD vaccines take effect as early as a few days after vaccination, exhibiting very unique capability to prevent chickens from the challenge of virulent MDV strains and MD lymphomas. Chicken macrophages and NK cells are activated after MDV vaccination and believed to participate in the innate immunity to MDV (45, 46). Indeed, we observed significant expansion of KUL01^+^ monocytes/macrophages but not NK cells in lung, spleen and blood (data not shown). Surprisingly, γδ T cells and CD8 T cells were also found to respond rapidly and significantly to CVI988 immunization (3 dpi), suggesting they may be participants of early immunity against MD. Despite a previous study that showed that γδ T cells increased in the spleen after very virulent MDV RB1B infection, we did not observe the same phenomenon, instead we noted a transient expansion of γδ T cells in the lung (Supplementary Figure 3), which was probably due to different experimental conditions. Thus, we believe that the expansion of γδ T cells is CVI988-specific in the context of the present study. Although the CVI988 vaccine was administrated intraperitoneally (not intranasally), it elicited the most robust and lasting expansion of γδ T cells and CD8 T cells in the lung, the primary site of MDV infection (Figures 2, 3). The immune response in the lung has long been overlooked in previous studies though the cytotoxicity of T lymphocytes and cytokine expression in the blood in most cases and in fewer cases in the spleens and cecal tonsils have been demonstrated (20, 21, 23, 32, 33, 41). Our results indicated that it is necessary and even more informative to examine the immune response in local tissues than in blood.

γδ T cells play important roles in host defense against many pathogens and tumors in mammals (27), For instance, γδ T cells provide protective immunity against herpes simplex virus type 1 and avian influenza virus infection (47, 48). They can secret a wide range of cytokines such as IFN-γ, TNF-α, IL-17, IL-21, and IL-22 and exert direct cytotoxicity to infected and transformed cells by release of granzymes and perforin (27). Although the cytotoxicity of CVI988-activated γδ T cells was not tested in the present study, it is plausible to speculate that these cells are able to kill target cells as chicken γδ T cells were shown to have spontaneous CTL activity before infection (29) and markedly upregulated the expression of granzyme, perforin, and NK lysin, the effector molecules of CTLs after CVI988 vaccination (Figure 7). However, a conclusive role of chicken γδ T cells in CVI988-induced immune protection remains to be addressed by depletion of this subset. Of note, as chicken γδ T cells dominantly express CD8α co-receptor, as shown in this study, the depletion of CD8 T cells with anti-chicken CD8α monoclonal antibody performed by Morimura et al. might also eliminate γδ T cells (24, 25), thus masking the contribution of γδ T cells to anti-viral immunity to MD. In contrast, using anti-chicken CD8αβ monoclonal antibody to deplete CD8 T cells resulted in partial CD8α^+^ T cells depletion leading to increased tumor incidence in only monovalent (SB1 or HVT) but not bivalent (SB1+HVT)-immunized chickens (26). These two studies implied that the CVI988 and SB1+HVT bivalent vaccination might elicit other anti-tumor mechanisms. It would be interesting to examine whether γδ T cells could contribute to such anti-tumor immunity.

In order to explain the protective mechanism of MD vaccines, the expression of cytokines including IFN-α, IFN-γ, IL-1β, IL-4, IL-6, IL-8, IL-12, IL-18, granzyme A, NK-lysin, perforin have been extensively detected after CVI988 immunization in previous studies (32, 33, 41), without any classification for the function of each cytokine in innate and adaptive immunity. As to which subset of immune cell expressed specific cytokines or effector molecules remained unclear. By cell sorting and specifically quantifying cytotoxic and effector T cell-associated cytokines, we clarified the sources of major effector molecules and their kinetic changes after immunization. The expression level and kinetics of granzyme A, perforin and IFN-γ in the leukocytes isolated from tissues (Figure 6) was consistent with previous reports in terms of fold change (32, 33). However, after cell sorting, we were able to detect higher level of expression for those cytokines on specific cell population (Figure 7). In line with the kinetic changes of CTL activity previously reported (23), the expression of cytotoxicity-associated granzyme A, perforin, NK lysin and IFN-γ all peaked at 7 dpi and was hardly detected at later time-points (14 and 21 dpi). However, through cell sorting, we could detect the expression of granzyme A and TNF-α at later time-points, suggesting that they may be indicators of adaptive immunity induced by CVI988. While IFN-γ has been suggested as a key factor in the protection against MD (32, 33), our results showed that granzyme A may be correlated with the protection due to its long-lasting expression after immunization. Of note, although chicken γδ T cells were able to express IL-17A (29), CVI988 vaccination did not upregulate its expression on this population in the present study. Unexpectedly, IL-2, a critical cytokine for T cell proliferation (49), was not upregulated after CVI988 immunization. A further study is needed to examine the role of IL-2 on the expansion of γδ T cells and CD8 T cells after CVI988 vaccination. Unlike previous findings that showed upregulation of IL-10 mRNA at 21 dpi after CVI988 vaccination (32) or virulent MDV RB1B infection (31), we observed that the mRNA expression of IL-10 was increased at early phase (3 and 7 dpi), concomitant with the increased expression of granzyme A, NK lysin and IFN-γ, in the tissues and sorted γδ T cells and CD8 T cells, indicating that IL-10 may play a role in balancing immune responses early after CVI988 immunization.

Virulent MDV and vaccine strains do not induce sterilizing immunity, leading to the persistence of MDV field viruses and vaccine strains (1, 11). Indeed, we can detect the CVI988 *meq* gene over the course of this study after immunization (Figure 1). This gives rise to a question whether CVI988-induced T cell response have memory response as persistent infection causes T cell exhaustion, characterized by progressive loss of T cell effector function, upregulated expression of inhibitory receptors and poor recall responses (50, 51). In mammals, memory T cells phenotypically express many surrogate markers including CD44, CD45RO, CD62L, and CD127 and display higher magnitude of secondary response after adoptive transfer of the primed T cells followed by challenge or re-stimulation with the same pathogen or antigens (52). While such immunological reagents for memory T cells are not yet available in chickens, we directly compared the primary and secondary T cell responses in naïve and previously immunized chickens to define the memory response. In addition, given that chickens have a high frequency of γδ T cells and the fact that memory γδ T cells have been identified in mouse, human (53, 54), and bovine (55), we specifically examined the recall response of chicken γδ T cells after reimmunization with CVI988. Our results demonstrated that CVI988-activated γδ T cells do not have memory response whereas CD8α^+^ T cells (TCRγδ^−^CD3^+^CD8α^+^) have recall response after secondary immunization with CVI988 (Figure 8).

In summary, through a comprehensive analysis of T cell response in CVI988-vaccinated chickens, we demonstrated that chicken γδ T cells and CD8α^+^ T cells significantly expanded at the early stage after immunization and displayed phenotypic and dynamic changes as well as differential cytokine expression. Although the vaccine viruses persist in chickens, CVI988 induced memory CD8α^+^ T cells but not γδ T cells. Our results, for the first time, demonstrated a potential role of γδ T cells in CVI988-induced early immune protection in chickens and provided further insights on the mechanism of immune protection against MD.

## Data Availability Statement

The original contributions presented in the study are included in the article/Supplementary Material, further inquiries can be directed to the corresponding authors.

## Ethics Statement

The animal study was reviewed and approved by Jiangsu Province Administrative Committee for Laboratory Animals.

## Author Contributions

SS and XH designed the experiment. XH, SL, JL, and YY performed experiments. XH analyzed data. AQ provided materials. SS and XH wrote the manuscript. All authors contributed to the final version of the manuscript.

## Conflict of Interest

The authors declare that the research was conducted in the absence of any commercial or financial relationships that could be construed as a potential conflict of interest.
