# Supplementary material for: An Anti-Tumor Vaccine Against Marek's Disease Virus Induces Differential Activation and Memory Response of γδ T Cells and CD8 T Cells in Chickens
Source: Front Immunol. 2021 Feb 15;12:645426. doi: 10.3389/fimmu.2021.645426 (PMC7917234; doi:10.3389/fimmu.2021.645426)

Supplementary Material

**Fig. S1. Gating strategies to define chicken T cell subsets and their co-receptor expression.** Mononuclear cells were isolated from spleens of 4 weeks-old naïve chicken and surface stained with antibody cocktails (CD3, TCRγδ, CD4, CD8α, CD8β and FVD eFluor 780). The lymphocytes are chosen with FSC versus SSC (A) and then singlets are gated using FSC-A and FSC-H (B). Live cells are defined as FVD eFluor 780-negative (C). By outputting CD3 versus TCRγδ, CD3^+^TCRγδ^+^ (γδ T cells), CD3^+^TCRγδ^-^ are identified, respectively (D). The γδ T cells contains CD8α^+^, CD4^+^ and CD4^-^CD8^-^ γδ T cells (E). CD3^+^TCRγδ^-^ T cells contain CD4^+^ (TCRγδ^-^CD3^+^CD4^+^) and CD8α^+^ T cells (TCRγδ^-^CD3^+^CD8α^+^) (G). Both γδ T cells and CD8α^+^ T cells can be further subdivided into CD8αα and CD8αβ subsets (F and H), respectively.

**Fig. S2. Representative dot-plots depict γδ T cells and TCRγδ^-^CD3^+^ T cells after CVI988 immunization.** Chickens were immunized with or without CVI988 (2000 PFU) and euthanized at indicated time-points, single cell suspensions from the spleen and blood were prepared for numerating γδ T cells by flow cytometry. Representative dot-plots depict γδ T cells and TCRγδ^-^CD3^+^ T cells in the spleens (A) and blood (B) of indicated birds at 3, 7, 14 and 21 dpi. Numbers indicate the percentages of γδ T and CD3^+^ T cells.

**Fig. S3. The dynamic changes of chicken γδ T cells and CD8^+^ T cells after MDV RB1B infection.** Chickens were infected with or without virulent MDV RB1B (1000 PFU) and euthanized at indicated time-points, single cell suspensions from the lung, spleen and blood were prepared for numerating γδ T and CD8^+^ T cells by flow cytometry. (A) and (B) Kinetic changes of the percentages of γδ T (A) and CD8^+^ T cells (B) in the lung and spleen of RB1B-infected and uninfected birds. Data shown are mean ± SD from 4 birds per group. **P*< 0.05.

**Fig. S4. The preferential proliferation of CD8α^+^ γδ T cells in spleen after CV988 vaccination.** Single cell suspensions were prepared from the control and CVI988-immunized chickens at indicated time-points and the dynamic changes of different subsets of γδ T cells (TCRγδ^+^CD3^+^) were analyzed by flow cytometry. (A) Representative dot-plots depict different subsets of γδ T cells (CD8^+^, CD4^+^ and CD4^-^CD8^-^) in the spleen of indicated birds at 3, 7, 14 and 21 dpi. Numbers represent the percentages of cells in each quadrant. (B) Kinetic changes of the percentage (upper panel) and number (lower panel) of CD8^+^ (left panel)and CD4^-^CD8^-^ γδ T cells (right panel) in the spleen of the control and CVI988-vaccinated chickens. Data shown are mean ± SD from six birds per group. **P*< 0.05, ***P*< 0.01, ****P*< 0.001.

**Fig. S5. The up-regulated expression of CD8αα co-receptor on chicken CD8α^+^ T cells after immunization.** Single cell suspensions were prepared from the lungs and spleens of mock- and CVI988-immunized chickens at indicated time-points, CD8α and CD8β expressions on TCRγδ^-^CD8α^+^ T cells were analyzed by flow cytometry. (A) Representative dot-plots depict CD8αβ^+^ and CD8αα^+^ (CD8α^+^CD8β^-^) CD8 T cells from indicated birds at 3, 7, 14, and 21 dpi. Numbers represent the percentages of cells in each quadrant. (B) and (C) Kinetic changes of the percentage of CD8αα^+^T cells in the lung (B) and spleen (C) of non-vaccinated and CVI988-vaccinated chickens at indicated time-points. (D) Representative histograms show CD8α expression on TCRγδ^-^CD8α^+^T cells from the lung and spleens of non-vaccinated and CVI988-vaccinated chickens at 3 and 7 dpi. (E) Bar graphs show MFI of CD8α on TCRγδ^-^CD8α^+^T cells from the two groups at 3 and 7 dpi. Data shown are mean ± SD from six birds per group. **P*< 0.05, ***P*< 0.01.

**Fig. S1**


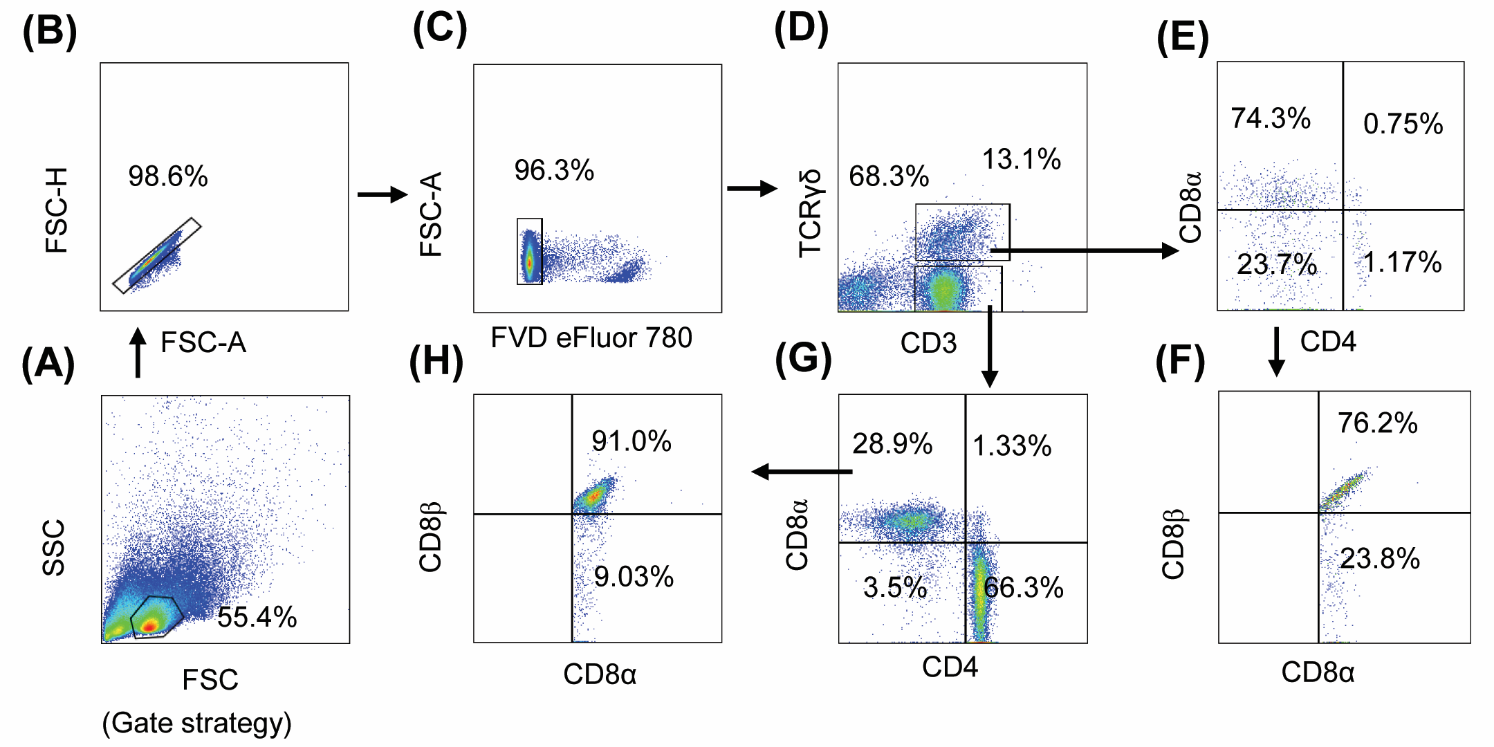


**Fig. S2**


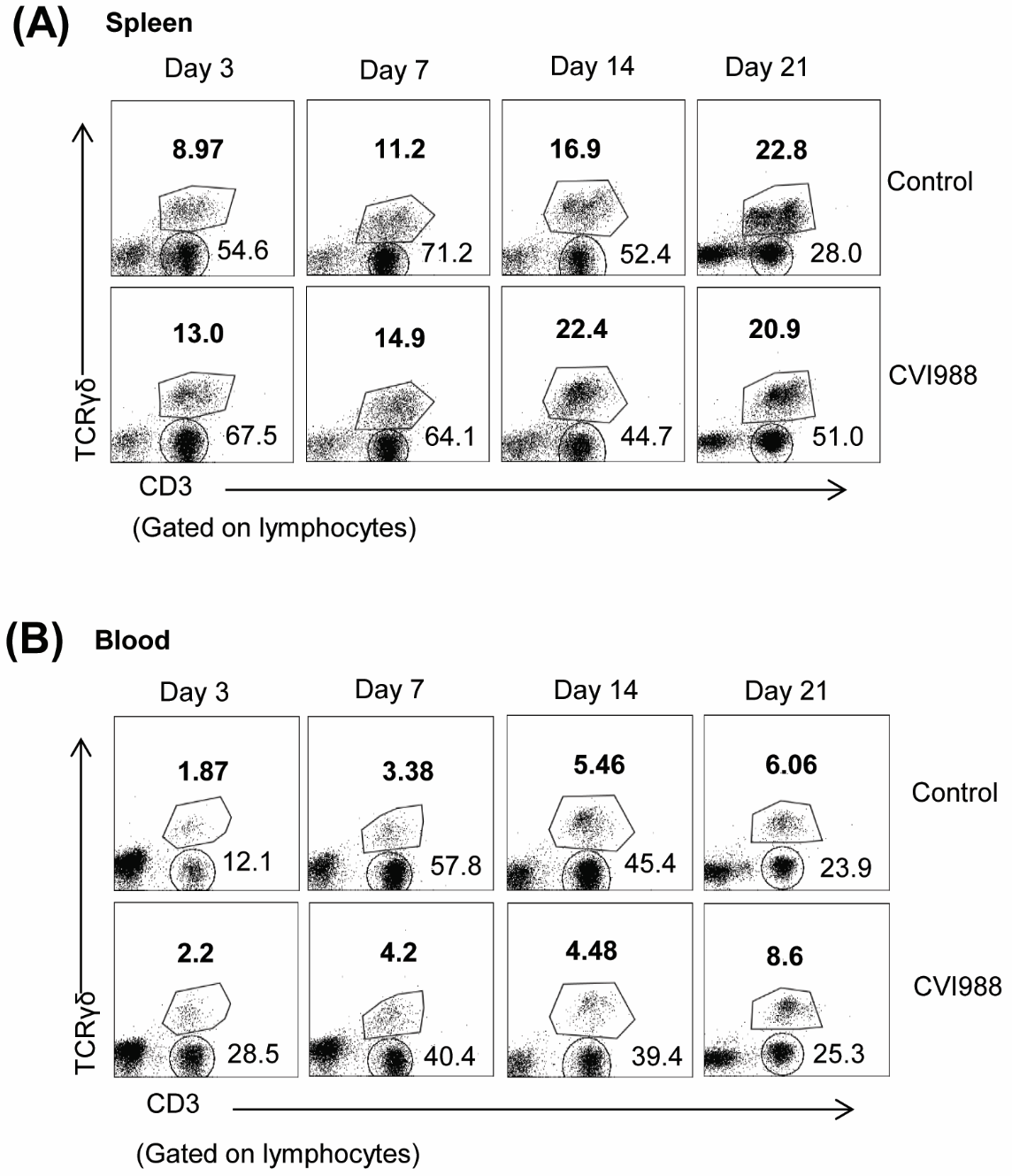


**Fig. S3**


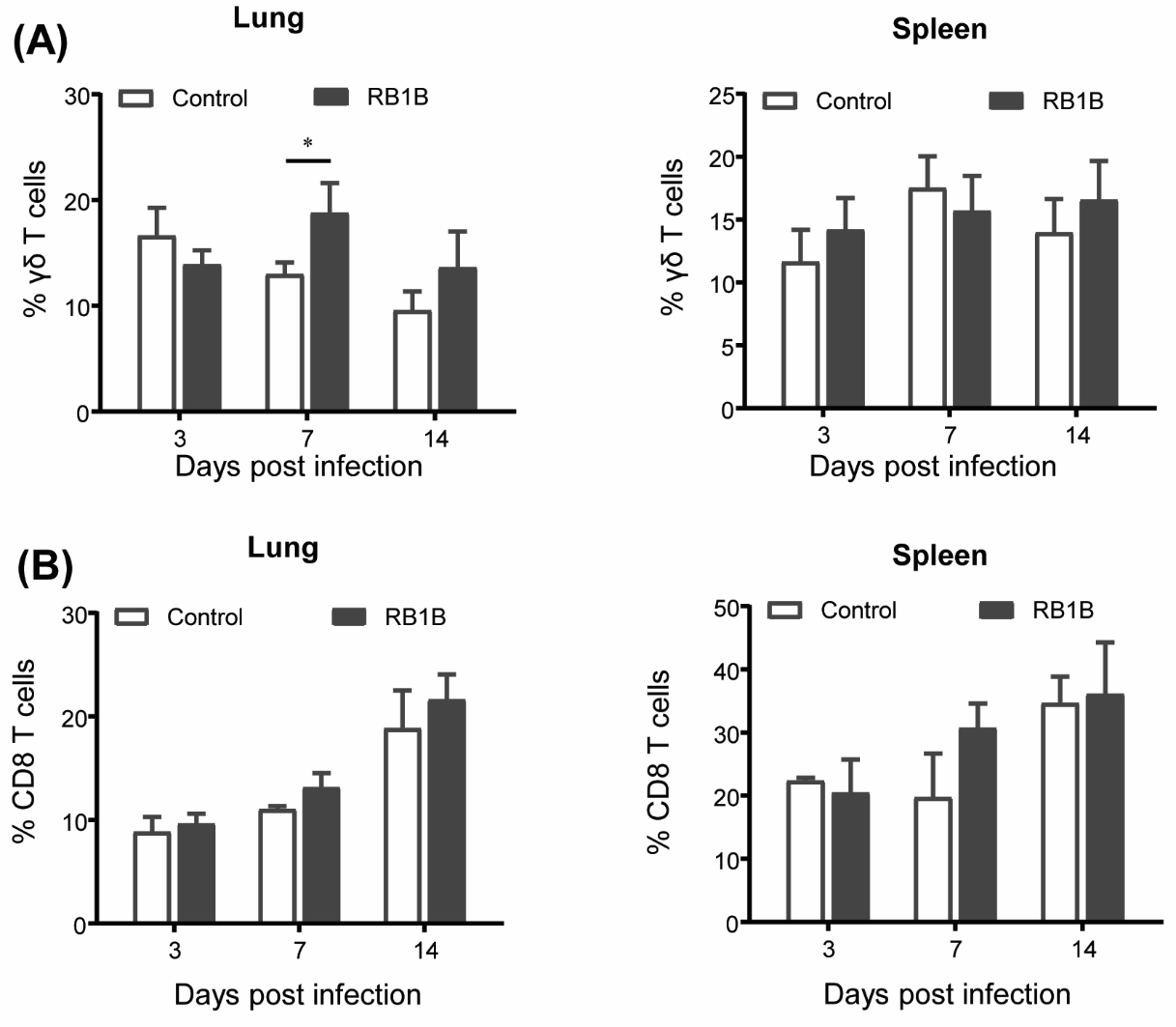


**Fig. S4**


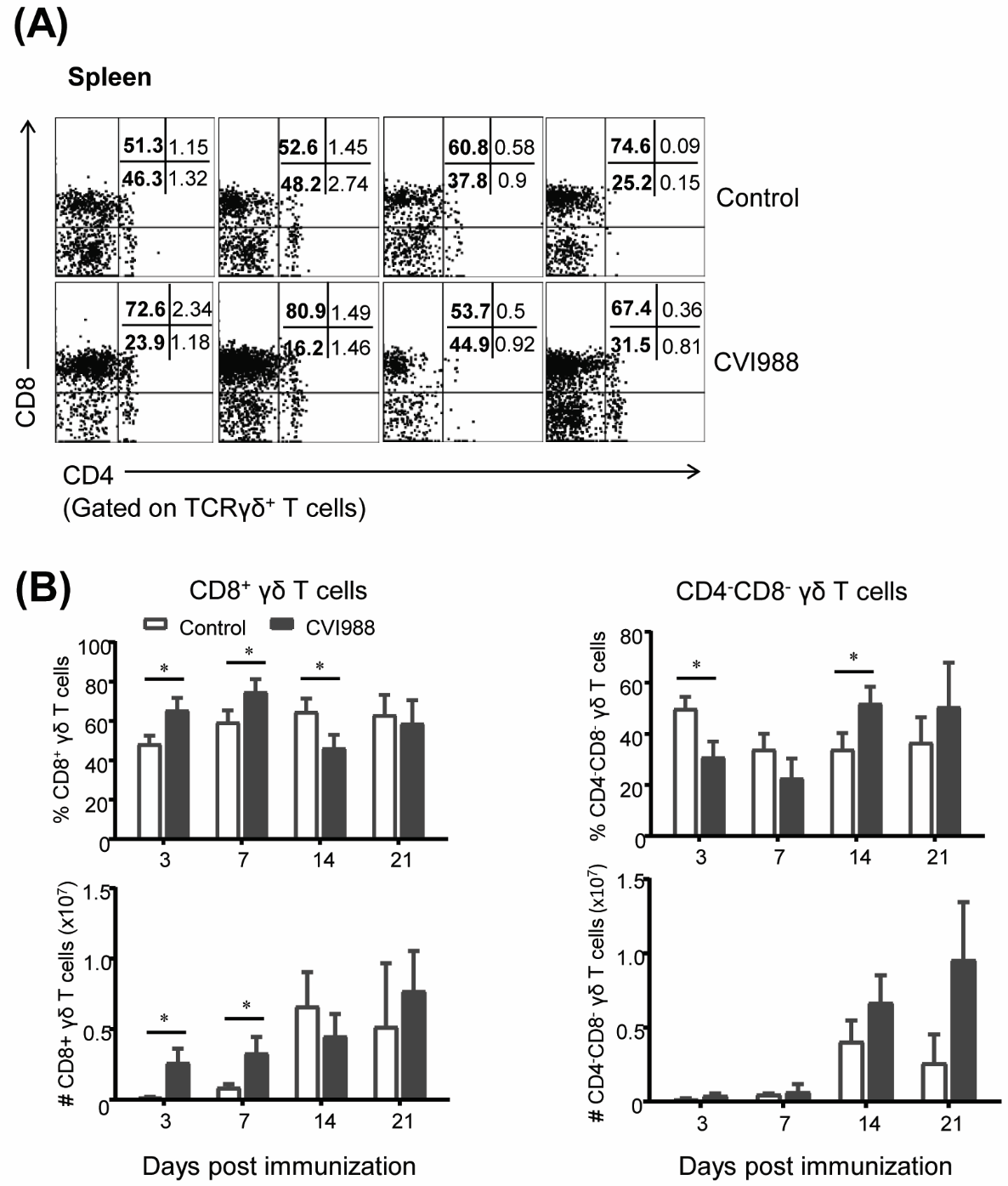


**Fig. S5**


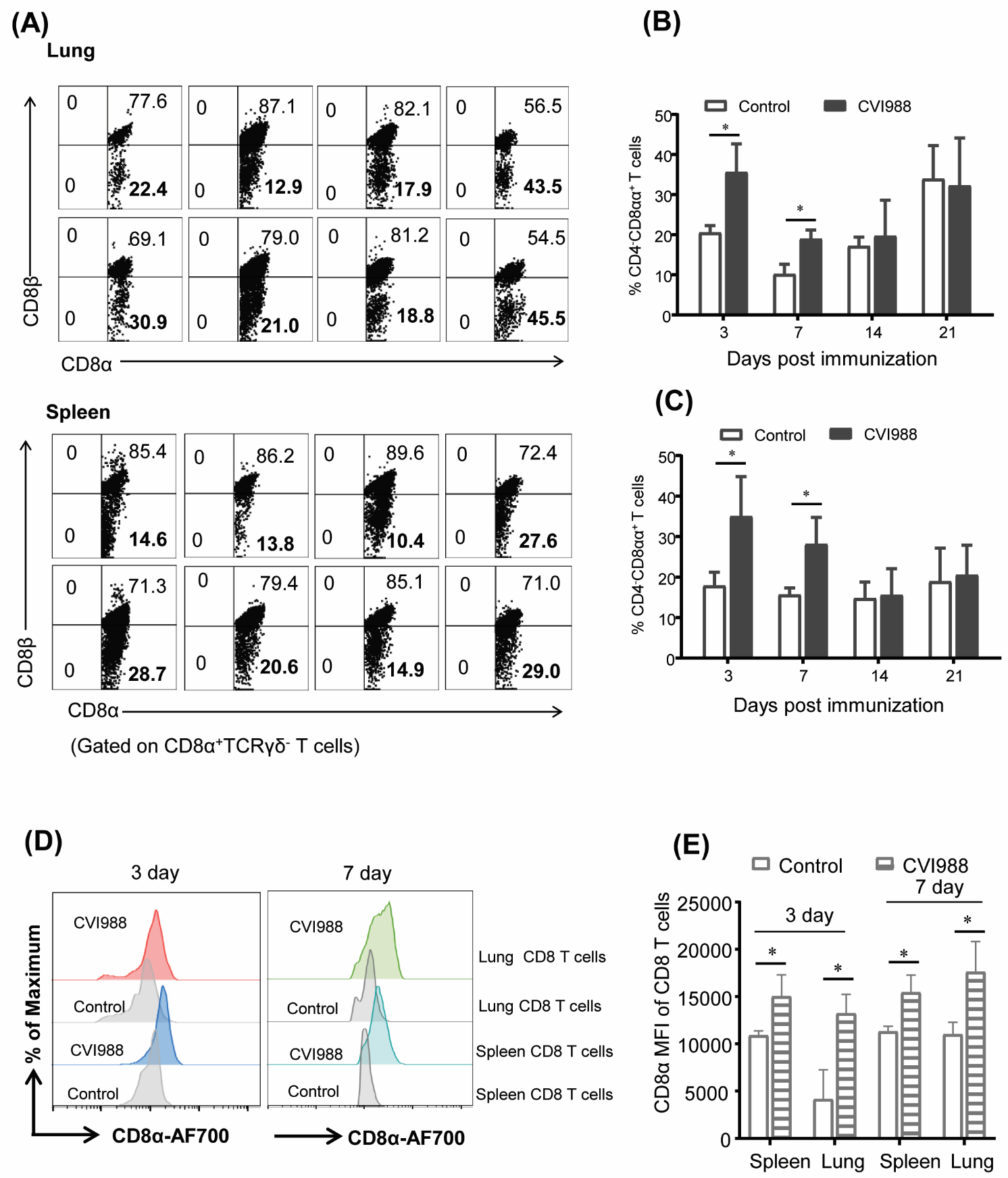

Supplement: Supplementary file 1 [file Data_Sheet_1.docx]
